# A Genetic Algorithm-Based Approach for Quantitative Prediction of Drug-Drug Interactions Caused by Cytochrome P450 3A Inhibition or Induction in Horses

**DOI:** 10.3390/ph19060815

**Published:** 2026-05-22

**Authors:** Veronica Di Paolo, Francesco Maria Ferrari, Italo Poggesi, Mauro Dacasto, Luigi Quintieri, Francesca Capolongo

**Affiliations:** 1 Department of Comparative Biomedicine and Food Science, University of Padua, Viale dell’Università 16, 35020 Legnaro, Italy; mauro.dacasto@unipd.it (M.D.); francesca.capolongo@unipd.it (F.C.); 2 Department of Research and Development, MeteRSit, 35129 Padua, Italy; fraul189@gmail.com; 3 Quantitative Clinical Pharmacology, GSK, 37135 Verona, Italy; italo.x.poggesi@gsk.com; 4 Laboratory of Drug Metabolism, Department of Pharmaceutical and Pharmacological Sciences, University of Padua, 35121 Padua, Italy; luigi.quintieri@unipd.it

**Keywords:** drug–drug interactions, cytochrome P450 3A, genetic algorithm, equine

## Abstract

**Introduction:** A genetic algorithm (GA)-based approach was designed to predict drug–drug interactions (DDIs) triggered by cytochrome P450 3A (CYP3A) inhibition or induction in horses. **Methods:** Area under the concentration-time curve ratios (AUCRs), obtained from published in vivo DDI studies in horses, were used to compute the following parameters: (1) the contribution ratio (CR), i.e., the fraction of the substrate dose metabolized via the CYP3A pathway, and (2) the interacting drug’s inhibitory potency or inducing efficacy (IR or IC, respectively). **Results:** AUCRs for 9 substrates, 12 inhibitors, and 1 inducer of equine CYP3A were predicted and validated with the developed method. More than 96% of predictions fell within the commonly accepted range of 50–200% of observed values. **Conclusions:** The proposed GA-based method may be a useful tool to estimate possible clinically relevant DDIs when co-administration of a CYP3A substrate and a CYP3A-interacting drug is anticipated.

## 1. Introduction

Therapeutic management of equines represents a significant aspect in both the breeding sector and equine sports, with implications that extend far beyond the mere cost of treatment. This field encompasses various categories of horses, including racehorses and those employed in equine-assisted therapy, each with its own specific economic and therapeutic considerations and requirements. In the context of horse racing, therapies constitute a crucial investment. These high-value animals require state-of-the-art medical care to maintain peak performance levels [1,2,3]. Costs can be substantially high, including specialized treatments, surgical interventions, and rehabilitation therapies [4,5]. However, these investments are often justified by the potential economic returns derived from competition victories and the animal’s breeding value [6]. For horses engaged in equine-assisted services (EAS), the impact of therapies is reflected not only on the health of the animal, but also on its ability to perform a therapeutic role [7]. EAS is a comprehensive term for all services in which professionals involve horses (or other equines) for the benefit of people. Therefore, maintaining the health of these horses is essential to ensure the continuity and efficacy of these programs. In this context, the monitoring and management of potential health issues, including those related to pharmacotherapy, become critical. The availability of new treatment options has increased the likelihood of equine patients being subjected to multiple drug regimens for the treatment of comorbid conditions [8]. Multidrug therapy in horses, as in human clinical practice, poses a considerable risk of drug–drug interactions (DDIs), in particular metabolism-based DDIs [9], with potential implications for treatment safety and efficacy [8]. In humans, the majority of metabolism-based DDIs are related to the inhibition or induction of proteins belonging to the cytochrome P450 (CYP) superfamily, and particularly CYP3A4, which is the enzyme most frequently involved in the phase I oxidative metabolism of xenobiotics [10]. CYPs exhibit marked species-specific differences in their expression patterns, catalytic efficiencies, regioselectivity, and inhibitor susceptibility profiles. Consequently, metabolic pathways and DDIs characterized in humans cannot be directly extrapolated to other species, including the horse, in the absence of species-specific data [8,11,12]. This concept is well illustrated by equine CYP3A subfamily members. Despite exhibiting a relatively high sequence identity to human CYP3A4 and CYP3A5 (79–86% for the coding sequence and 68–81% for the amino acid sequence) [13], they display functional properties that diverge substantially from those of human CYP3A enzymes. For example, equine CYP3A94, CYP3A95, and CYP3A97 have been shown to metabolize testosterone primarily to 2-hydroxytestosterone, reflecting a markedly different regioselectivity compared with human CYP3A enzymes [14]. This represents a significant metabolic divergence from humans, where 6β-hydroxytestosterone is the predominant testosterone metabolite produced by the primary adult human CYP3As, i.e., CYP3A4 and CYP3A5 [15]. In addition, CYP3A96, which, among the characterized equine CYP3A enzymes, most closely resembles human CYP3A4 based on its catalytic behavior [16,17] and its dual hepatic-intestinal expression pattern [18], exhibits a markedly reduced catalytic capacity toward nifedipine, metabolizing it approximately ten-fold more slowly than CYP3A4 [16].

Further highlighting these interspecies differences, the response of equine CYP3As to prototypical CYP3A inhibitors diverges markedly from that observed in humans. In particular, ketoconazole potently inhibits CYP3A95, whereas troleandomycin reduces the activity of both CYP3A94 and CYP3A95. Conversely, neither compound exerts significant effects on CYP3A89-catalyzed reactions [19].

Although CYP3A induction is a well-defined mechanism of metabolism-based DDIs in humans, based on the available literature, no studies have directly quantified CYP3A induction in horses, leaving the magnitude and enzyme specificity of such effects unknown. In this context, drugs used in horses and known to result in clinically relevant metabolism-based DDIs in other species include both well-known human CYP3A inducers, such as phenobarbital and rifampicin, as well as known human CYP3A inhibitors, e.g., ketoconazole, itraconazole and cimetidine [8]. However, the absence of species-specific induction data, combined with the documented functional divergence of equine CYP3A members, makes it impossible to extrapolate inducer effects from humans to horses with confidence.

Collectively, the marked interspecies differences in CYP3A catalytic behavior and inhibitor sensitivity suggest that the propensity for CYP3A-mediated DDIs might differ substantially between horses and humans. In particular, the divergent inhibitor susceptibility profiles observed among equine CYP3A enzymes in vitro [19] indicate that prototypical CYP3A inhibitors characterized in humans might not elicit comparable modulatory effects in horses, further complicating the extrapolation of interaction risk across species.

Despite the clinical relevance of DDIs in equine medicine, published data on metabolism-based DDIs in horses remain scarce. The available evidence includes in vivo pharmacokinetic studies reporting AUCR values for interactions involving commonly used equine drugs. In the absence of comprehensive species-specific metabolic profiling data, the attribution of these drugs as CYP3A substrates in the horse was based on analogy with their well-characterized role as CYP3A substrates in humans, an approach justified by the functional similarities between equine and human CYP3A enzymes, particularly CYP3A96, which most closely resembles human CYP3A4 based on its catalytic behavior and dual hepatic-intestinal expression pattern [16,17]. These studies, while limited in number and heterogeneous in design, provide the only available species-specific data for the quantitative characterization of CYP3A-mediated DDIs in horses, and represent the empirical foundation of the present study.

In human pharmacology, several methodological frameworks have been developed for the quantitative prediction of CYP-mediated DDIs. Among these, the Ohno method has emerged as a particularly valuable approach, based exclusively on in vivo pharmacokinetic data and requiring minimal experimental input. Originally developed for the prediction of CYP3A4-mediated DDIs in humans [20,21], this method has been successfully extended to other CYP-mediated pathways, including CYP1A2, CYP2C8, CYP2C19, CYP2D6, and CYP2B6 [22,23,24,25], demonstrating its versatility across different metabolic pathways. More recently, the approach has been translated to veterinary species, with successful applications reported for CYP-mediated DDIs in dogs and cats [26].

This study aims to apply and validate the Ohno method, optimized through a genetic algorithm, for the quantitative prediction of CYP3A-mediated DDIs in horses. Specifically, the study seeks to: (a) estimate in vivo parameters representing the fractional contribution of CYP3A to the overall metabolic clearance of substrate drugs (CR), the inhibition ratio of inhibitor drugs (IR), and the induction coefficient (IC) for commonly used equine drugs; (b) validate the predictive performance of the framework against an independent dataset of published AUCR values; and (c) demonstrate the practical utility of the approach for predicting the magnitude of DDIs for novel substrate-interacting drug combinations not yet available in the published literature, thereby providing a practical tool for DDI risk assessment in equine clinical practice when more sophisticated approaches such as PBPK modeling cannot be applied due to the scarcity of resources, time, or species-specific data.

## 2. Results

**Figure 1:**
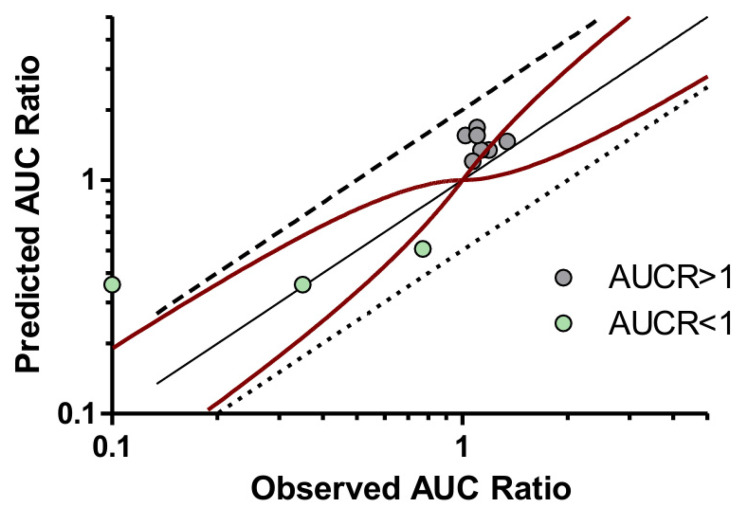
External Validation: Predicted versus observed area under the concentration-time curve ratios (AUCRs) used for prediction of CYP3A-mediated drug–drug interactions (DDIs) in horses. Solid red curves denote intervals as suggested by Guest et al. [27]. The solid gray line is the identity line (y = x). The top and the lower dashed lines represent y = 2x and y = 0.5x, respectively. AUC: area under the plasma concentration-time curve.

The analysis encompassed a total of 26 peer-reviewed clinical studies, as listed in Table 3. Fifteen of these were used to predict CR, IR, and IC parameters in Step 1, whereas the remaining 11 were used to validate the method in Step 2 through external validation, as detailed in the Section 4. The validation process involved plotting the predicted AUCR values against observed AUCR values (Figure 1). The model demonstrated reliable performance, with 90% of the predicted AUCR values falling within 50–200% of the observed AUCR values. The predictive reliability of the method was further supported by comparison with the Guest intervals, with most predicted AUCR values falling within, or very close to, the Guest limits, as shown in Figure 1.

**Figure 2:**
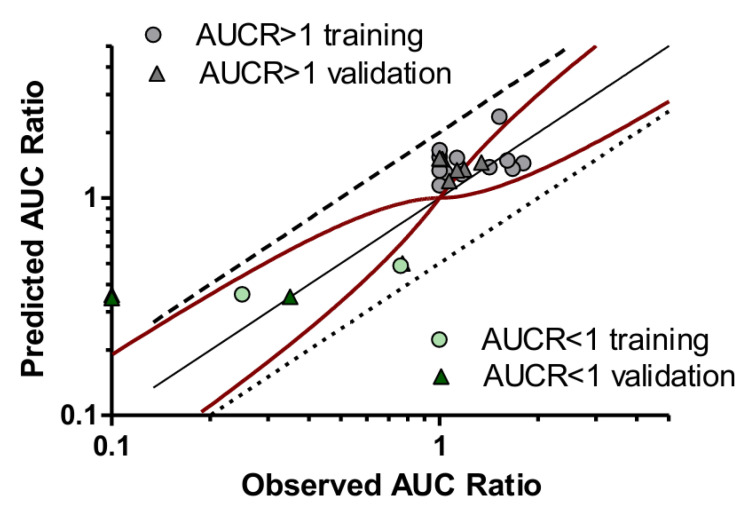
Predicted versus observed area under the concentration-time curve ratios (AUCRs) with final estimates of CYP3A contribution ratio (CRs), CYP3A inhibition ratio (IRs) and CYP3A increases in drug clearance (ICs) in horses. The triangle symbols are predictions from the external validation step; the filled circles are the predictions from the final estimation step. The red solid curves denote the limits as suggested by Guest et al. [27]. The grey solid line is the line of identity (y = x). The upper and lower dashed lines represent y = 2x and y = 0.5x, respectively. AUCR training: drug–drug interaction (DDI) data used for initial estimation of CRs, IRs and ICs; AUCR validation: DDI data used for external validation. Linear regression analyses led to the following equation: y = 0.9103x + 0.2798. AUC: area under the plasma concentration-time curve. AUCR: AUC ratio.

Table 1 and Table 2 show the final Bayesian orthogonal regression estimates obtained, along with their 95% confidence intervals, for putative equine CYP3A substrates, inhibitors, and inducers. The relationships between predicted AUCR and observed AUCR values corresponding to each CYP3A substrate-inhibitor/inducer drug pair are plotted in Figure 2.

Data presented in Table 1 and Table 2 could be used in clinical practice to predict the extent of novel, yet unknown, CYP3A-based DDIs in horses. In particular, by substituting the final estimates into Equations (1) and (2), the safety of the co-administration of a CYP3A substrate drug and a CYP3A inhibitor or inducer in horses can be assessed.

**Figure 3:**
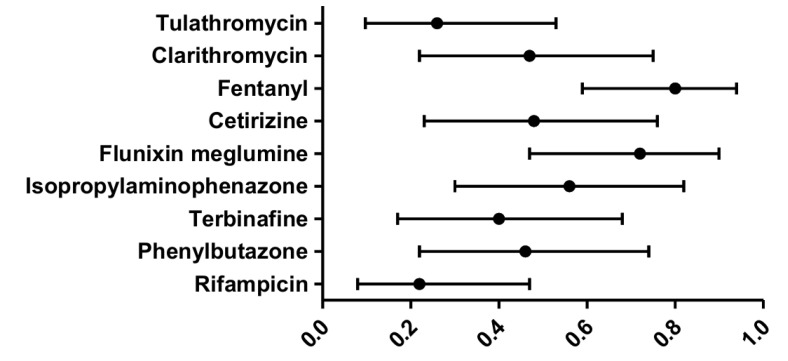
Forest plot of estimated CR values for the nine substrate drugs included in the analysis. Points represent the parameter estimates; horizontal lines represent the 95% credible intervals derived from the Bayesian analysis.

**Figure 4:**
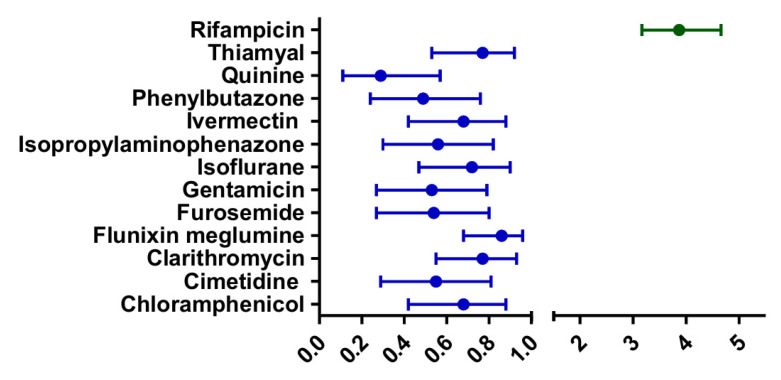
Forest plot of estimated IR values for the twelve inhibitors and IC value for the inducer included in the analysis. Points represent parameter estimates; horizontal lines represent the 95% credible intervals derived from the Bayesian analysis. Inhibitory ratios (IR) and induction coefficient (IC) are shown in different colours.

The estimated CR values with their corresponding 95% credible intervals are illustrated in Figure 3. The CR values ranged from 0 to 1, indicating variability in the extent to which the selected substrate drugs depend on CYP3A-mediated metabolism. The estimated IR and IC values with their corresponding 95% credible intervals are shown in Figure 4. IR values ranged from 0 to 1, reflecting differences in the inhibitory potency of the inhibitor drugs on equine CYP3A. The IC value for rifampicin was 3.87, indicating a moderate induction of CYP3A-mediated metabolism. The width of the credible intervals reflects the degree of uncertainty associated with each parameter estimate, which is influenced by the number of AUCR observations available for each drug.

## 3. Discussion

The present study provides a GA-based approach to predict DDIs putatively involving CYP3A in horses, addressing a significant gap in veterinary pharmacology. GA is widely employed as a search and optimization technique for addressing highly complex problems. The fundamental concept of GA is inspired by the natural evolutionary processes observed in living ecosystems, aimed at modeling biological variables as solutions to the problem at hand [28]. In GA, each potential solution within the population is evaluated using a fitness function, which serves as an objective measure of its quality. The algorithm then employs a stochastic selection mechanism to identify superior solutions, with the probability of selection being proportional to the fitness value. This approach favors better solutions while still allowing for the occasional selection of less optimal ones.

The inclusion of a probability for selecting suboptimal solutions is a key feature that helps GA avoid becoming trapped in local optima. This characteristic enables the algorithm to potentially escape from local maxima or minima, as subsequent iterations may introduce variations that lead to improved global solutions. Thus, even if high-quality solutions are temporarily caught in a local optimum, the inherent variability in the selection process provides opportunities for discovering better solutions in unexplored regions of the search space [28].

The validation of our model, with 90% of predicted AUCR values falling within the acceptable range of 50–200% of observed AUCR values, demonstrates its potential as a useful tool for veterinary medicine. Model performance was quantitatively assessed using the Geometric Mean Fold Error (GMFE) and the Mean Absolute Percentage Error (MAPE). The GMFE, which is considered the most appropriate metric for PK prediction data given their inherently asymmetric distribution, yielded a value of 1.39 on the overall dataset (n = 26) and 1.41 on the validation set (n = 10). MAPE was 45.6% and 49.3% for the overall and validation datasets, respectively. The Consistency between overall and validation metrics further supports the absence of overfitting and model robustness.

Unlike in human medicine, where extensive clinical trials precede drug approval and marketing, drug treatment in horses frequently relies on pharmacological data extrapolated from other species. Our approach represents a method to predict metabolism-based DDIs in horses, potentially improving safety and efficacy in clinical practice.

The practical utility of this model lies in its ability to predict AUCR values for drug combinations that have not yet been studied clinically in horses. This could be particularly valuable for veterinarians when considering the use of multiple drugs in horse patients, especially in cases of comorbidities or complex treatment regimens.

It is worth noting the discrepancies observed in the literature regarding the rifampicin-clarithromycin interaction. Among the studies listed in Table 3, three report AUCR values for this drug combination, but with conflicting results. Two studies report an AUCR of 0.1, while a third study by Berlin et al. [29] reports a value of 0.35. However, the studies reporting the lower AUCR of 0.1 present certain limitations, including either high standard deviations associated with the determination of pharmacokinetic parameters or a small sample size of animals used in the study.

Interestingly, the prediction generated by our model aligns more closely with the AUCR value of 0.35 reported by Berlin et al. [29], whose study employed 12 Thoroughbred foals, providing a more robust sample size. This alignment lends credibility to the predictive capability of our approach. Nevertheless, the presence of such discordant values in the literature inevitably results in some predicted values falling outside the typical acceptability range. This situation underscores the complexity of drug interactions in equine pharmacology and highlights the need for careful interpretation of both literature data and model predictions. It should be noted that the Ohno method evaluates DDIs exclusively through CYP3A-mediated mechanisms and does not account for transporter-mediated interactions or the parallel involvement of other CYP enzymes. In the case of rifampicin, which is a pleiotropic inducer known to affect CYP3A, drug transporters, and intestinal absorption, these alternative mechanisms may contribute to the observed DDIs in ways not fully captured by the current model. This represents an inherent limitation of simplified DDI prediction approaches, whose main advantage lies in their applicability when PBPK models are unavailable. Although dose-dependent IR estimates would theoretically be preferable, the limited availability of veterinary DDI data precluded stratification by dose or treatment regimen, as this would have resulted in an insufficient number of observations for meaningful validation. A single IR value per inhibitor was therefore assigned as a pragmatic approach consistent with the constraints of the available dataset. The selection of substrate drugs as equine CYP3A probes was based on analogy with human CYP3A data, reflecting the current scarcity of species-specific experimental evidence in the horse. Among the characterized equine CYP3A enzymes, CYP3A96 most closely resembles human CYP3A4 based on its catalytic behavior and dual hepatic-intestinal expression pattern, representing the equine isoform that is most closely functionally analogous to the human enzyme. This represents an inherent limitation of the present analysis, which could be addressed in future studies employing equine hepatic microsomes or recombinant equine CYP3A enzymes. The present framework treats the equine CYP3A subfamily as a single composite pathway, consistent with the original Ohno method [26,30]. Although this simplification may obscure isoform-selective inhibition and induction, as illustrated by the markedly different responses of individual equine CYP3A enzymes to prototypical inhibitors, a hierarchical multi-isoform framework is currently precluded by the limited availability of isoform-specific pharmacokinetic data in the horse. The assumption that CR values are invariant across inhibitors or inducers represents an inherent simplification of the Ohno method, consistent with its original formulation [20,21] and subsequent extensions [22,23,24,25]. In practice, inhibitors may affect parallel metabolic pathways, protein binding, or hepatic blood flow, potentially altering the apparent CR value. These effects are not captured by the current framework, which is designed to provide practical DDI predictions with minimal data requirements rather than to fully characterize the mechanistic complexity of drug interactions.

However, it is important to acknowledge the additional limitations of this study. While the proposed approach demonstrates good predictive ability, it is based on a relatively small number of DDIs observed in vivo. Although these in vivo studies provide valuable real-world data, the limited number of DDIs examined may not fully represent the wide range of potential interactions in equine pharmacology. To further assess model robustness and minimize the risk of overfitting, validation predictions were evaluated by splitting the dataset into training and validation datasets, the latter of which was used to establish the predictive ability of the approach. Further validation with additional drug combinations and larger sample sizes would strengthen the model’s reliability and broaden its applicability, currently limited by the limited knowledge base of in vivo DDI studies in horses. It should be noted that a minority of CR and IR parameters are informed by a single AUCR observation only. Although the global optimization GA approach adopted and the network structure of CR-IR/IC combinations support overall parameter identifiability, individual parameter estimates should be interpreted with caution. Two approximate AUCR values (≈1 and ≈0.1) were treated as their corresponding numerical values in the model. The value of ≈1 reflects the absence of a clinically relevant drug–drug interaction, thus carrying negligible uncertainty. Regarding the value of ≈0.1, which indicates a significant induction effect, the limited number of animals used in the original studies did not allow additional significant figures to be reported. Given that small numerical differences carry disproportionate weight at such low values (e.g., 0.10 vs. 0.15 represents a 50% numerical difference while corresponding to a comparable degree of induction), the value of 0.1 was deliberately adopted as a conservative worst-case estimate. Moreover, it is crucial to consider that factors such as individual variability, breed differences, and disease states could influence DDIs in ways not fully captured by the current model. The heterogeneity represents another inherent limitation of the pooled dataset, reflecting the critical scarcity of veterinary DDI data available in the literature compared to human pharmacokinetic studies, which are typically conducted under controlled conditions in healthy volunteers. Since data are reported as group means rather than individual animal values, formal stratification by breed, age, or clinical condition could not be performed. These factors may introduce complexities that are challenging to account for in a generalized predictive model, highlighting the need for cautious interpretation and application of the results in clinical settings. The development of a Bayesian hierarchical meta-analytic framework incorporating study-level covariates such as breed, age, formulation, and dosing protocol represents an important direction for future research. Such an approach is currently precluded by the unavailability of individual animal pharmacokinetic data and the limited number of studies available in the veterinary literature, which would provide insufficient statistical power to reliably estimate covariate effects as additional model parameters. The static deterministic equations of the Ohno method do not capture the time-dependent nature of induction and mechanism-based inhibition, which depend on enzyme turnover rates and progressive CYP inactivation, respectively. A dynamic turnover or physiologically based framework would provide a more mechanistically complete description of these processes, but would require substantially more species-specific experimental data than are currently available for equine CYP3A enzymes. This represents an important direction for future methodological refinement.

To illustrate the practical application of the proposed approach, we present a worked example involving the combination of phenylbutazone (a CYP3A substrate) and clarithromycin (a CYP3A inhibitor), for which no published AUCR data are currently available in the equine literature. Using the CR estimate for phenylbutazone (CR = 0.46; CrI: 0.22–0.74) and the IR estimate for clarithromycin (IR = 0.77; CI: 0.55–0.93), the predicted AUCR was calculated by applying Equation (1):

AUCR = 1/(1 − CR × IR) = 1/(1 − 0.46 × 0.77) = 1/0.646 = 1.55

The clinical interpretation of AUCR predictions should consider the therapeutic window of the substrate drug. For CYP3A inhibition, AUCR values below 2 are generally associated with modest clinical risk, although monitoring may be warranted for drugs with a narrow therapeutic index. For CYP3A induction, AUCR values below 0.5 should be regarded as clinically significant, reflecting a reduction in substrate exposure exceeding 50% that may compromise therapeutic efficacy and potentially result in therapeutic failure, warranting dose adjustment or alternative therapeutic strategies.

Although PBPK modeling would provide a more mechanistically complete framework for DDI prediction, its application to the horse is currently limited by the scarcity of species-specific physiological and biochemical data required for model parameterization. The Ohno method represents a practical and accessible alternative, particularly suited to equine clinical practice where PBPK modeling expertise and resources are rarely available. Future studies should explore the development of equine PBPK models as species-specific data become increasingly available.

In conclusion, while our approach is not intended to replace clinical judgment or eliminate the need for careful monitoring, it provides a useful tool to guide decision-making in equine pharmacology.

In conclusion, this study presents the first application of the Ohno method to the prediction of CYP3A-mediated DDIs in horses. The proposed framework estimated CR, IR, and IC parameters for 9 substrate drugs, 12 inhibitors, and 1 inducer, with acceptable predictive performance as indicated by a GMFE of 1.39 and MAPE of 45.6% on the overall dataset, and GMFE of 1.41 and MAPE of 49.3% on the independent validation set. All predictions met the Ohno acceptability criterion, with 60% of validation predictions additionally falling within or in close proximity to the Guest intervals.

## 4. Materials and Methods

A framework was established and applied to predict DDIs elicited by putative inhibitors or inducers of equine CYP3A. A similar approach was recently applied by our research group to the prediction of metabolism-based DDIs in humans [30], dogs, and cats [26]. Details of the rationale and demonstration of the equations presented below have been provided in the original publications by Ohno et al. [20,21], who first developed the approach for the prediction of DDIs in humans caused by inhibition or induction of human CYP3A4. The use of the same equations has been extended by other research groups to the prediction of other CYP-mediated DDIs in humans (see, e.g., [22,23,24,25]).

For DDIs caused by inhibition of one or more equine CYP3A enzymes, the area under the concentration-time curve ratio (AUCR) is calculated from Equation (1):

(1) $$\frac{{AUC}^{*}}{AUC}=\frac{1}{1\;-\;CR\;\times IR}$$

where *AUC* denotes the systemic exposure to the CYP3A substrate when given as monotherapy, and AUC* reflects the systemic exposure to the substrate drug upon concurrent administration of a CYP3A inhibitor. *CR* is defined as the contribution ratio (i.e., the fraction of the substrate clearance due to metabolism via CYP3A), and *IR* is the inhibition ratio for the inhibitor. *IR*, representing the in vivo potency of the inhibitor integrated over time, depends on both the dose of the inhibitor and its inhibition constant (*K*_i_). Both *CR* and *IR* are positive numbers, ranging from 0 to 1. *CR* equals 0 when CYP3A makes no contribution to the metabolic clearance of the CYP3A substrate drug, while it is 1 when one or more CYP3A enzymes are entirely responsible for its metabolism. *IR* is equal to 0 when the interacting drug gives rise to no inhibition, and 1 when the inhibitor fully suppresses CYP3A activity. Consequently, the AUCR for inhibition ranges from 1 (indicating no inhibitory effect) to infinity (complete inhibition of substrate drug elimination).

For DDIs resulting from the induction of one or more equine CYP3A, the AUCR is obtained using Equation (2):

(2) $$\frac{AUC*}{AUC}\;=\;\frac{1}{1\;+\;CR\;\times IC}$$

where *AUC* is the AUC of the substrate drug when administered alone, and AUC* is the AUC of the substrate drug when co-administered with a CYP3A inducer. As in Equation (1), *CR* is the contribution ratio, and *IC* is the apparent increase in clearance of the substrate drug caused by CYP3A induction. *IC* quantifies the inducer efficacy, ranges from 0 to any positive value, and depends on both the dose and efficacy of the inducer, as well as the duration of inducer treatment [21]. In this scenario, the AUCR ranges between 0 and 1, where 1 indicates no enzyme induction.

The AUCRs used in our prediction model, listed in Table 3, were retrieved from DDI studies conducted on healthy equines published in peer-reviewed journals listed in PubMed and/or Google Scholar.

**Table 3:** Studies used for Step 1 (initial estimation of model parameters using GA) or Step 2 (external validation) of drug–drug interactions mediated by CYP3A inhibition or induction in horses.

Inhibitor	Interacting Drug		Substrate	Step	Observed AUCR	Reference
	Dose mg/kg	Treatment				
Chloramphenicol	50.0	TID 1 D	Phenylbutazone	Step 1	1.80	[31]
Chloramphenicol	33.0	1 D	Phenylbutazone	Step 2	1.34	[32]
Cimetidine	4.0	14 D	Phenylbutazone	Step 2	1.19	[33]
Cimetidine	4.3	1 W	Phenylbutazone	Step 2	1.13	[34]
Cimetidine	30.0	1 D	Terbinafine	Step 1	1.17	[35]
Clarithromycin	7.5	5 D	Rifampicin	Step 1	1.05	[36]
Clarithromycin	7.5	BID 3 D	Rifampicin	Step 2	1.07	[37]
Flunixin meglumine	1.1	1 D	Phenylbutazone	Step 1	≈1	[38]
Flunixin meglumine	1.1	1 D	Phenylbutazone	Step 2	≈1	[39]
Furosemide	1.1	1 D	Phenylbutazone	Step 1	≈1	[40]
Gentamicin	2.2	3 D	Phenylbutazone	Step 1	1.04	[41]
Isoflurane	3.3	1 D	Fentanyl	Step 1	1.52	[42]
Isopropylaminophenazone	12.0	1 D	Phenylbutazone	Step 1	1.67	[43]
Ivermectin	0.2	1 D	Cetirizine	Step 1	1.61	[44]
Phenylbutazone	3.50–4.50	2 D	Flunixin meglumine	Step 2	1.02	[45]
Phenylbutazone	6.0	1 D	Isopropylaminophenazone	Step 1	1.42	[43]
Phenylbutazone	2.2	1 D	Flunixin meglumine	Step 1	≈1	[38]
Phenylbutazone	4.4	1 D	Flunixin meglumine	Step 2	≈1	[39]
Quinine	20.0	1 D	Phenylbutazone	Step 1	≈1	[32]
Thiamyal	11	1 D	Phenylbutazone	Step 1	1.13	[46]
**Inducer**	**Interacting Drug**		**Substrate**	**Step**	**Observed AUCR**	**Reference**
	**Dose** **mg/kg**	**Treatment**				
Rifampicin	10.0	BID 13 D	Clarithromycin	Step 2	0.35	[29]
Rifampicin	10.0	BID 2 D	Clarithromycin	Step 1	0.25	[47]
Rifampicin	10.0	BID 1 D	Tulathromycin	Step 2	0.77	[48]
Rifampicin	10.0	8 D	Tulathromycin	Step 1	0.76	[48]
Rifampicin	10.0	BID 3 D	Clarithromycin	Step 2	≈0.1	[37]
Rifampicin	10.0	BID 11 D	Clarithromycin	Step 2	≈0.1	[36]

The proposed approach consisted of three stages: (1) initial estimation of model parameters using a genetic algorithm (GA); (2) external validation of predicted values; and (3) final estimation of CR, IR, and IC values through a Bayesian orthogonal regression.

### 4.1. Step 1: Initial Estimation of Model Parameters Using GA

GA mimics the process of natural selection in evolution to solve the global optimization problem [49]. During Step 1, GA was initialized with stochastic CR, IR, and IC values using MATLAB software, version R2024b (MathWorks Inc., Natick, MA, USA). The constraints used to guide the GA in the initial estimation of CR and IR values were calculated by plotting Equations (1) and (2). Further details of the constraints used are available in our previous paper, where a similar GA-based approach was used to predict DDI involving human CYP2C8 or human CYP2B6 [30]. For the prediction of DDIs by GA, 16 AUCRs were available, related to 9 substrates (9 CRs), 12 inhibitors (12 IRs) and 1 inducer (1 IC). Thus, the vectors of decision variables were [CR1, …, CR9, IR1, …, IR12, IC1].

**Figure 5:**
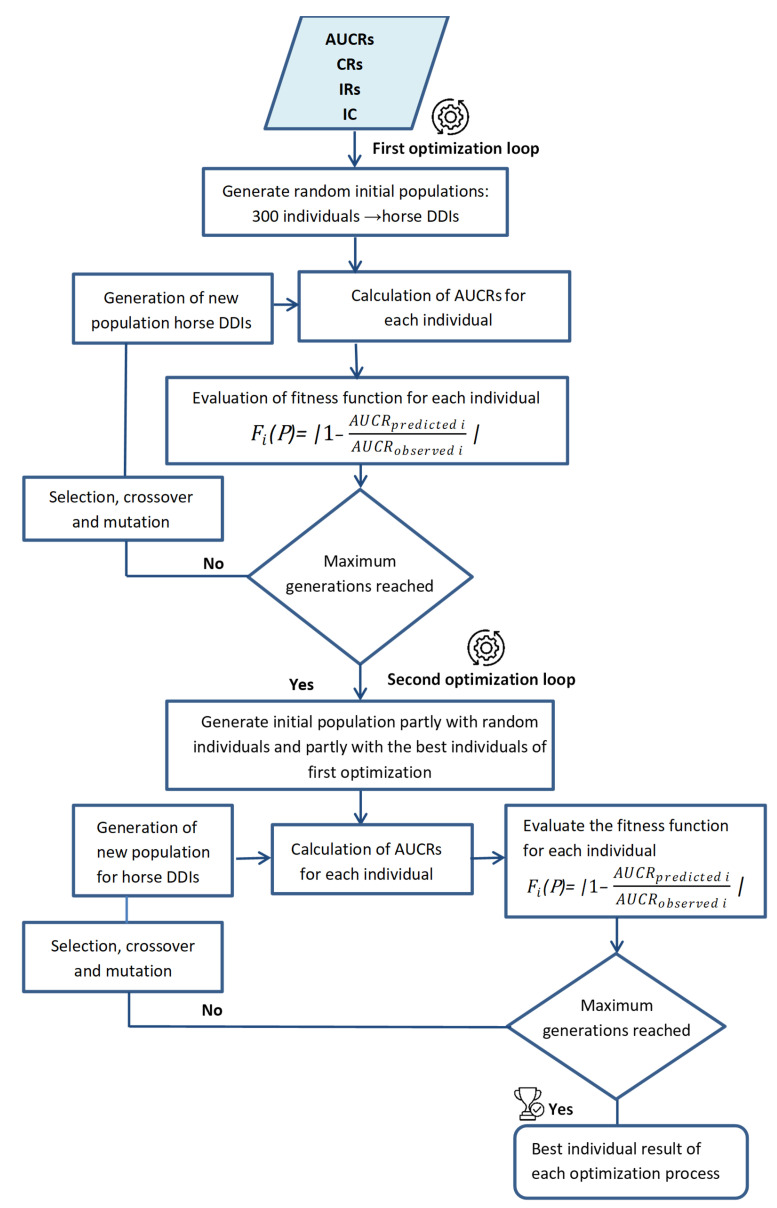
Genetic Algorithm Flowchart.

The optimization process of the multi-object problem aimed to minimize the following function:

$$F_{i}(P)\;=\;|1\;-\;\frac{{AUCR}_{predicted\;i}}{{AUCR}_{observed\;i}}|$$

where $F_{i}$ is the fitness function for each ${AUCR}_{predicted\;i}$ used for Step 1 listed in Table 3, and P is the vector of decision variables. The optimization objective is to determine the values of decision variables that minimize the difference between the experimentally observed AUCR (${AUCR}_{observed\;}$) and the computationally derived AUCR (${AUCR}_{predicted\;}$) for each AUCR value considered in Step 1. The adopted GA parameters were: (a) population size per generation in the optimization iteration: 300 individuals; (b) maximum generation: 200 individuals; (c) crossover probability: 0.8 for the initial round and 0.9 for the subsequent round. The workflow of the GA methodology is summarized in Figure 5.

### 4.2. Step 2: External Validation of Predicted Values

To validate the predictions obtained by GA, 10 out of the 26 AUCRs reported in Table 3 were used for the external validation phase. Predicted AUCR values were obtained by solving Equations (1) and (2), using CR, IR, and IC values generated by the GA in Step 1. Subsequently, predicted AUCRs were compared with observed AUCRs via visual inspection of plots. The predictions were considered correct if 90% of predicted AUCR values fell within the 50–200% range of observed AUCR values [20,21]. Moreover, to verify the success of the prediction method, the predicted AUCR values were evaluated against the revised acceptance limits introduced by Guest et al. [27]. Prediction error (i.e., the difference between the predicted and observed value) and the absolute mean of the prediction error were also calculated to evaluate the imprecision of the prediction.

**Figure 6:**
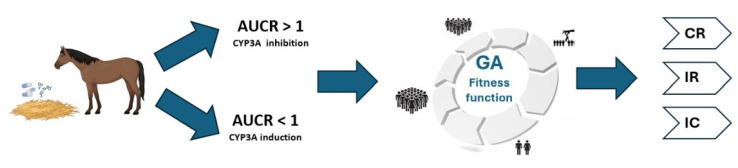
Graphical summary of the genetic algorithm (GA)-based approach for the prediction of drug–drug interactions (DDIs) caused by an equine CYP3A-interacting drug. The figure illustrates the predictive framework where AUCR (AUC ratio) values greater than 1 indicate CYP3A inhibition, while values less than 1 indicate CYP3A induction. The parameters CR (concentration ratio), IR (inhibition ratio), and IC (increase in drug clearance) are derived through an optimization process using GA, which iteratively optimizes the fitness function to achieve the most accurate prediction of DDI outcomes.

The overall predictive framework adopted in this study is schematically illustrated in Figure 6.

### 4.3. Step 3: Refined Estimation of CR, IR, and IC Values via Bayesian Orthogonal Regression

The final estimates of all model parameters were derived through Bayesian orthogonal regression using the WinBUGS software (version 1.4.3), as previously reported by Di Paolo et al. [26].

For each CYP3A substrate (σ) and inhibitor (κ), the predicted AUCR was implemented and coded in WinBUGS according to Equation (3):

(3) $${pred}_{\sigma \kappa }=\;\frac{1}{\left(1\;-\;{CRZ}_{\sigma \;}\times \;{IRZ}_{\kappa }\right)}$$

$${AUCratio}_{\sigma \kappa }\sim N({pred}_{\sigma \kappa },tauAUC)$$

where ${pred}_{\sigma \kappa }$ and ${AUCratio}_{\sigma \kappa }$ are the predicted and observed AUCRs, respectively, whereas ${CRZ}_{\sigma }$ and ${IRZ}_{\kappa }$ are the Bayesian posterior values (final estimates) of CR and I, respectively; *tauAUC* is the precision (i.e., the reciprocal of the variance) of the AUC distribution. A Gaussian distribution was assumed for AUCRs, whereas a logistic distribution with values ranging between 0 and 1 was set for both the CR and IR values. The mean of each distribution corresponded to the initial estimation obtained by GA in Step 1. The precision of these distributions was assumed as a gamma distribution: tauCR~(4,1); tauIR~*G*(4,1); tauAUC~*G*(2,1). Gamma distributions were set so that the expected standard errors of CR and IR values on the logit scale and of AUCRs were 0.5 and 5, respectively.

The CR values obtained from the inhibition data were subsequently used for the Bayesian regression analysis of the final estimates for DDI caused by CYP3A induction.

For each CYP3A substrate (σ) and inducer (j), the predicted AUCR was coded in WinBUGS according to Equation (4):

(4) $${pred}_{\sigma \eta }=\;\frac{1}{\left(1\;+\;{CRZ}_{\sigma }\;\times \;\;{ICZ}_{j}\right)}$$

*AUCratio*_σj_~*N* (*pred*_σj_, *tauAUC*)

where *pred*_σj_ and *AUCratio*_σj_ are the predicted and observed AUCR for each CR and IC pair, respectively, while ICZ_j_ is the Bayesian posterior value (final estimation) of ICs. IC values are positive real numbers; thus, a log-normal distribution was assumed. As for CYP3A inhibition-based DDIs, the precision of the distribution was as a Gamma distribution tauAUC~*G* (2,1). The Gaussian likelihood was applied on the log-transformed scale for all parameters, which is mathematically equivalent to assuming a log-normal distribution on the original scale, thereby appropriately accounting for the positive and potentially skewed nature of AUCR data. The posterior distributions of all the estimated parameters (i.e., CRs, IRs, ICs, and AUCRs) were obtained through Monte Carlo Markov chain simulation (WinBUGS software). The means of posterior distributions were regarded as point estimations of CR, IR, IC and AUCR values with 95% confidence intervals. Convergence was assessed by visual examination of trace and history plots of parameter sample values versus the number of iterations, following the approach described by Gabriel et al. (2016) [25]. The precision of posterior estimates was evaluated using the Monte Carlo error provided by WinBUGS, with a Monte Carlo error of less than 5% of the sample standard deviation considered as the acceptability criterion. Credible intervals (CI) around the GA-derived parameter estimates were computed using WinBUGS. For IR and CR parameters, a logit-normal model was adopted with Gamma (4,1) priors on precision, given their bounded range [0, 1]. For the IC parameter, a log-normal model was specified with a weakly informative Gamma (1,0.002) prior on precision, reflecting its unbounded positive range. For all parameters, initial values were set to the corresponding GA-derived estimates, ensuring that chains started in a region of high posterior probability. All models were run for 5000 iterations with default WinBUGS settings (burn-in: 1000, thinning: 1). Finally, to assess the prediction success, all predicted AUCRs were plotted versus observed AUCRs and compared with the Guest intervals [27].

## Figures and Tables

**Table 1:** Refined parameters of contribution ratios (*CR*) of CYP3A substrates.

Substrate	*CR*	95% CI
Rifampicin	0.22	0.079–0.47
Phenylbutazone	0.46	0.22–0.74
Terbinafine	0.40	0.17–0.68
Isopropylaminophenazone	0.56	0.30–0.82
Flunixin meglumine	0.72	0.47–0.90
Cetirizine	0.48	0.23–0.76
Fentanyl	0.80	0.59–0.94
Clarithromycin	0.47	0.22–0.75
Tulathromycin	0.26	0.097–0.53

CI: confidence interval.

**Table 2:** Refined inhibition ratios (*IR*) of CYP3A inhibitors and increase in drug clearance (*IC*) of CYP3A inducer.

Inhibitor	Interacting Drug		*IR*	95% CI
	Dose mg/kg	Treatment		
Chloramphenicol	33.0–50.0	1 D TID	0.68	0.42–0.88
Cimetidine	4.0–30.0	1–14 D	0.55	0.29–0.81
Clarithromycin	7.5	3–5 D	0.77	0.55–0.93
Flunixin meglumine	1.1	1 D	0.86	0.68–0.96
Furosemide	1.1	1 D	0.54	0.27–0.80
Gentamicin	2.2	3 D	0.53	0.27–0.79
Isoflurane	3.3	1 D	0.72	0.47–0.90
Isopropylaminophenazone	12.0	1 D	0.56	0.30–0.82
Ivermectin	0.2	1 D	0.68	0.42–0.88
Phenylbutazone	2.2–6.0	1–2 D	0.49	0.24–0.76
Quinine	20.0	1 D	0.29	0.11–0.57
Thiamyal	11.0	1 D	0.77	0.53–0.92
**Inducer**	**Interacting Drug**		***IC***	**95% CI**
	**Dose**	**Treatment**		
Rifampicin	10.0	1–13 D	3.87	3.17–4.66

CI: confidence interval.

## Data Availability

The original contributions presented in this study are included in the article. Further inquiries can be directed to the corresponding author.
